# Stimulus-Specific Individual Differences in Holistic Perception of Mooney Faces

**DOI:** 10.3389/fpsyg.2020.585921

**Published:** 2020-11-06

**Authors:** Teresa Canas-Bajo, David Whitney

**Affiliations:** ^1^ Vision Science Graduate Group, University of California, Berkeley, Berkeley, CA, United States; ^2^ Department of Psychology, University of California, Berkeley, Berkeley, CA, United States; ^3^ Helen Wills Neuroscience Institute, University of California, Berkeley, Berkeley, CA, United States

**Keywords:** holistic perception, face perception, Mooney faces, individual differences, inversion effect

## Abstract

Humans perceive faces holistically rather than as a set of separate features. Previous work demonstrates that some individuals are better at this holistic type of processing than others. Here, we show that there are unique individual differences in holistic processing of specific Mooney faces. We operationalized the increased difficulty of recognizing a face when inverted compared to upright as a measure of the degree to which individual Mooney faces were processed holistically by individual observers. Our results show that Mooney faces vary considerably in the extent to which they tap into holistic processing; some Mooney faces require holistic processing more than others. Importantly, there is little between-subject agreement about which faces are processed holistically; specific faces that are processed holistically by one observer are not by other observers. Essentially, what counts as holistic for one person is unique to that particular observer. Interestingly, we found that the per-face, per-observer differences in face discrimination only occurred for harder Mooney faces that required relatively more holistic processing. These findings suggest that holistic processing of hard Mooney faces depends on a particular observer’s experience whereas processing of easier, cartoon-like Mooney faces can proceed universally for everyone. Future work using Mooney faces in perception research should take these stimulus-specific individual differences into account to best isolate holistic processing.

## Introduction

Humans perceive faces holistically rather than as a set of separate features (Sergent, 1984), and it is our ability to perceive faces as a whole that makes humans experts in face processing (Farah et al., 1998; Maurer et al., 2002). One of the strongest pieces of evidence for holistic perception of faces is the inversion effect (Yin, 1969); holistic processing breaks down with inverted faces (Sergent, 1984; Farah et al., 1995; Rossion, 2008). This results in reduced performance (lower accuracies and longer reaction times) when identifying inverted faces compared to upright faces (Yin, 1969; McKone, 2004; Busey and Vanderkolk, 2005), but there is no such drop in accuracy for inverted single features (McKone, 2004). Metrics like the composite-face test (McKone, 2008) and the whole-part test (Donnelly and Davidoff, 1999) provide further empirical evidence of holistic processing of faces.

**Figure 1:**
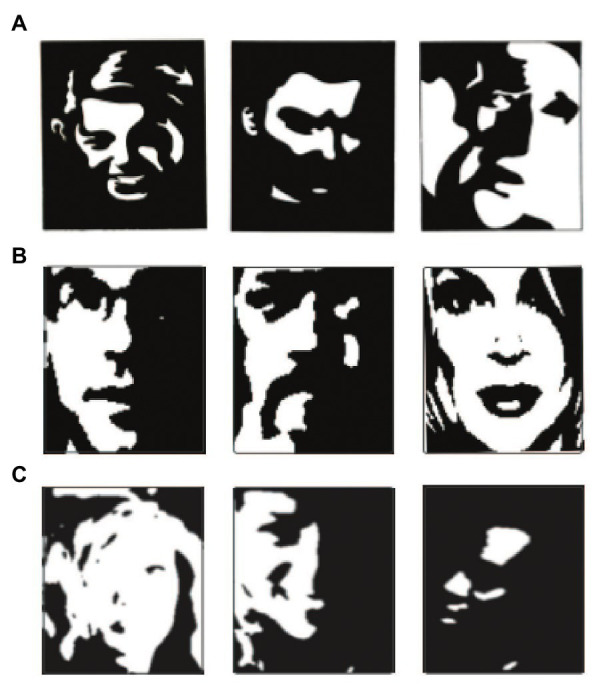
Example of three Mooney face datasets. **(A)** Mooney faces adapted from Mooney (1957). **(B)** Mooney faces created by a generative adversarial network model adapted from Ke et al., 2017. **(C)** Mooney faces created from thresholds of grayscale faces adapted from Schwiedrzik et al., 2018.

Studies of holistic processing often use the inversion effect as an operational measure of “holistic-ness” (McKone, 2004; Taubert et al., 2011) and frequently use Mooney faces as stimuli (Andrews and Schluppeck, 2004; George et al., 2005; Latinus and Taylor, 2005; Leo and Simion, 2009; Verhallen et al., 2014). Mooney faces (Figure 1) are two-tone black and white blobs that are readily perceived as faces despite lacking low-level segmentable face-specific features (Mooney, 1957). Mooney face recognition is all-or-none, either you perceive it as a face in a glance or it remains just an abstract impression of blobs (Brodski et al., 2015; Schwiedrzik et al., 2018), much like Dallenbach’s cow (Dallenbach, 1951) and Gregory’s Dalmatian dog (Gregory, 1970). Inversion of a Mooney face makes its identification almost impossible﻿ (Kanwisher et al., 1998; McKone, 2004; Latinus and Taylor, 2005). Because the inversion effect is stronger for Mooney faces than for gray-scale faces (McKone, 2004), they are often treated as a more efficient means of isolating holistic face processing.

There are a large variety of Mooney faces that have been created and published, including the original artist-created images (Mooney, 1957), thresholded images of gray-scale faces (Brodski et al., 2015; Goold and Meng, 2016; Schwiedrzik et al., 2018), and artificially created images based on machine learning approaches (Ke et al., 2017). While, Mooney faces are typically treated as an excellent stimulus to isolate holistic processing, there are also significant variations between different Mooney faces (Figure 1). This raises the question of whether different Mooney faces may be more or less effective at isolating the holistic mechanisms of face recognition.

Here, we measured holistic processing of individual Mooney faces using the magnitude of the inversion effect, a method widely accepted as a marker of holistic processing (Yin, 1969; McKone and Yovel, 2009; Taubert et al., 2011). Previous work has demonstrated that there are individual differences in holistic processing showing that some individuals are better at this holistic type of processing than others (Russell et al., 2009; Wang et al., 2012). Unlike previous studies, our approach measures the strength of the inversion effect for individual Mooney faces to identify the magnitude of holistic processing for specific stimuli and for specific observers. If there are consistent face-specific differences, it would suggest that individual face images should be evaluated before assuming that they tap into “holistic” processing. Additionally, there may be individual subject differences that interact with the individual faces: the particular faces that are holistic for one subject may or may not be the same as those for another subject. Assessing holistic processing within any given observer may require tailoring the stimuli to that subject’s unique fingerprint of face processing.

## Experiment 1: Per-Face Per-Observer Individual Differences

### Method

#### Participants

Thirty participants took part in this experiment. All subjects were undergraduate students at the University of California, Berkeley and provided written consent form before participation. All experimental procedures were approved by the UC Berkeley Institutional Review Board.

#### Materials

The stimuli consisted of 192 images (96 ambiguous Mooney faces and 96 shuffled Mooney stimuli). Literally thousands of Mooney faces have been created and published in the past (Brodski et al., 2015; Goold and Meng, 2016; Ke et al., 2017; Schwiedrzik et al., 2018). In the present study, we used a subset of Mooney faces created by Schwiedrzik et al. (2018) because these Mooney faces have been used to study individual differences, and because these stimuli include a diverse array of faces (varying in perspective, lighting direction, face identity, gender, etc.). It is worth noting that this data set is unique in that the scrambled stimuli were created manually from each respective Mooney face so it would preserve the main structure of both upright and inverted versions. The scrambled stimuli were modified in an iterative process in which each shuffled stimulus was tested separately until 85% of the subjects agreed that there was “no face” in it (see Schwiedrzik et al., 2018 for more controls and details). Each Mooney face had only one scrambled stimulus, so that the pair always appeared together. A CRT monitor at 60 Hz was used to display all the stimuli. The monitor was placed at 60 cm from the participant and at this distance Mooney stimuli subtended a visual angle of 10°. A chin rest was used to stabilize subjects head and distance to the monitor. The presentation of the stimuli was controlled using MATLAB R2016b with Psychophysics Toolbox 3 (Brainard, 1997; Kleiner et al., 2007).

#### Design

Each subject completed a total of 1,920 trials [2 (Upright vs. Inverted) × 96 Mooney stimuli × 10 repetitions of each Mooney Face]. The faces were repeated 10 times in each condition (upright and inverted) to generate enough trials to facilitate a split-half within subjects’ correlation procedure and increase the power of its results.

#### Procedure

**Figure 2:**
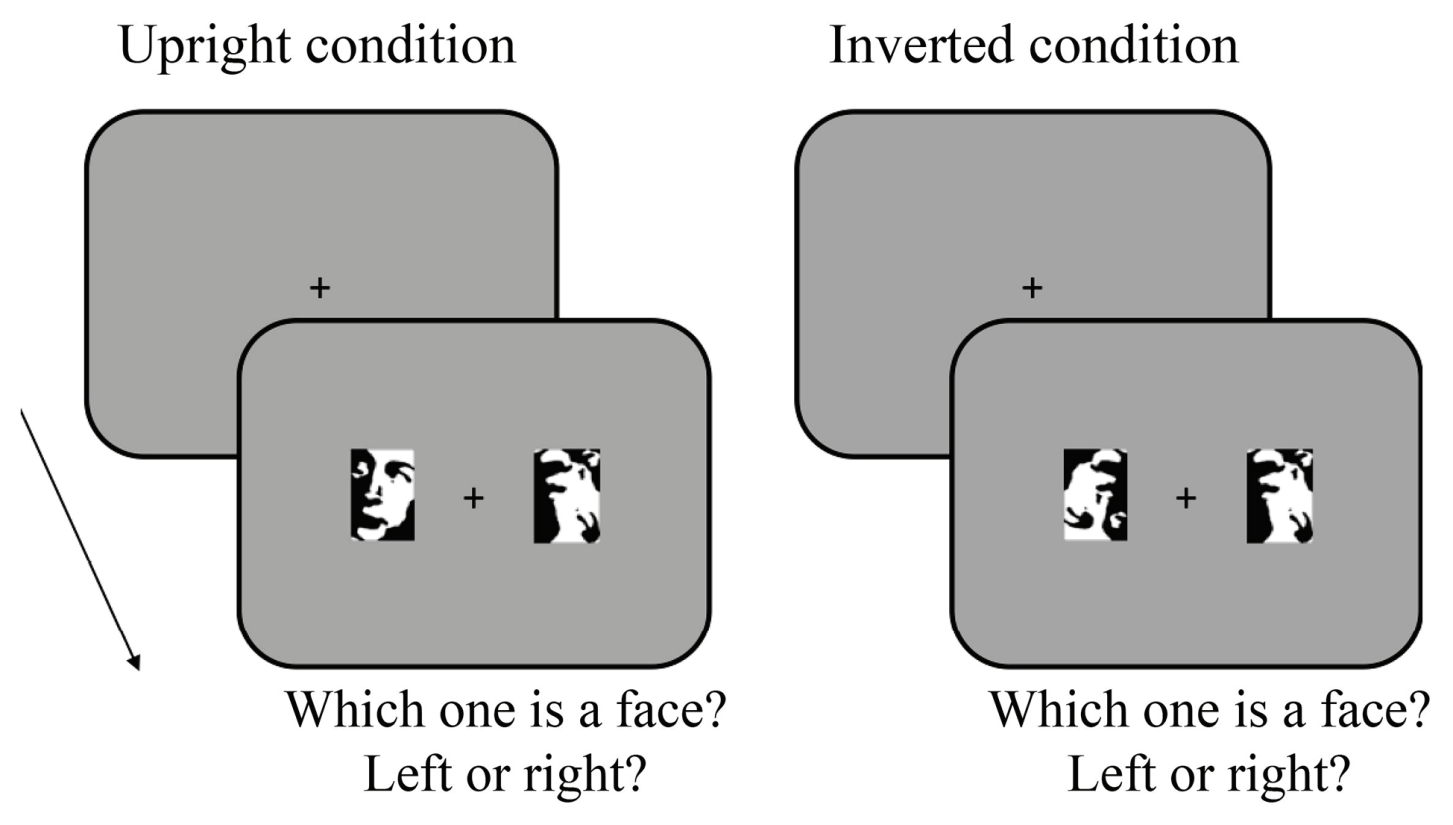
Task in Experiment 1 for the upright condition (left) and inverted condition (right). In both conditions, subjects had to first fixate a cross at the center of the screen. Then, two images appeared at either side of the screen, left or right. The observers’ task was to discriminate which of the two images was a face, by pressing the left or right arrow.

The task was to report which of two images was a face (left or right). Participants were instructed to ignore the orientation of the face (i.e., an upside-down face could be the correct answer). Each trial started with a fixation cross in the center of the screen displayed for 1 sec. Immediately after, two Mooney images were displayed, a Mooney face and a scrambled Mooney stimulus, one to the left of the fixation cross and the other one to the right of the fixation cross (Figure 2). The location of each stimulus was randomly chosen to be left or right in each trial. The orientation of the Mooney face was upright on half of the trials and inverted on the other half. The two conditions were randomly interleaved between trials. The two images were shown until a response was given (self-paced). Subjects were instructed to give their answers as fast as possible. After the stimuli onset, participants were allowed to free-view the two images.

### Data Analysis

The magnitude of the inversion effect for each Mooney face was calculated per subject by subtracting the average accuracy of that Mooney face in the upright condition and the average accuracy of that Mooney face in the inverted condition.

For all analyses, we used a bootstrap re-sampling procedure ﻿(Efron and Tibshirani, 1994). In each iteration of this procedure, the trial information used to calculate the average for upright and inverted conditions per subject, per Mooney face, was re-sampled with replacement for only five of the 10 trials available for each subject, face, and condition. The reason to only choose half of the trials was to allow for a split-half procedure to quantify within-subject consistency. To generate the null distributions for the within‐ and between-subject correlations, we shuffled the label of the Mooney face number and then bootstrapped the correlation without replacement.

### Results

**Figure 3:**
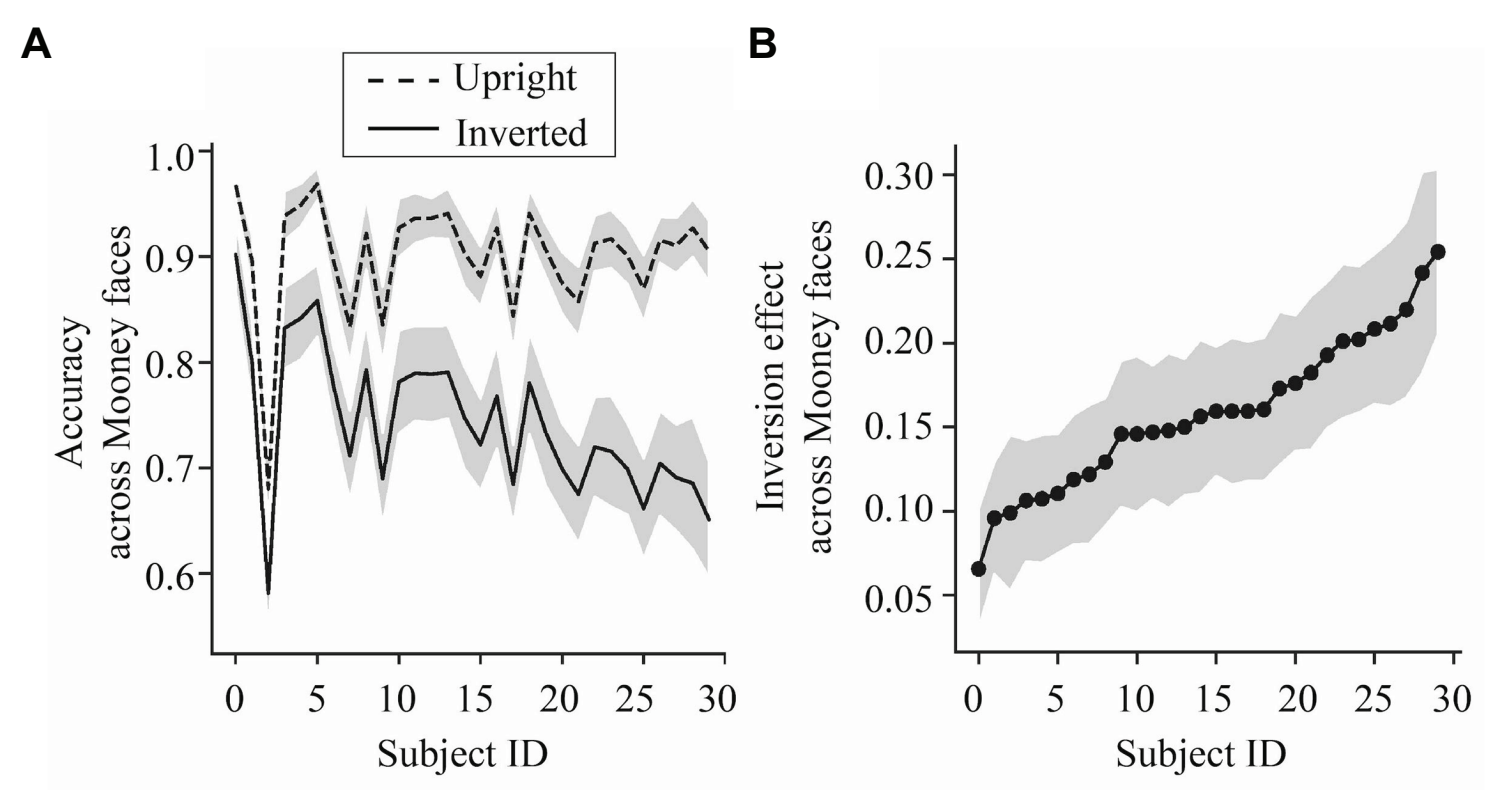
Results of Experiment 1. **(A)** Upright Mooney face (dashed) and inverted Mooney face (solid) discrimination accuracy (ordinate), averaged across all Mooney faces, for each subject (abscissa). The shaded region represents the 95% CIs around the mean performance for each subject. **(B)** Magnitude of the inversion effect (the difference between average accuracy in the upright condition and average accuracy in the inverted condition) for each subject (abscissa). Participants (subject ID) are ordered from those with weaker inversion effects to those with stronger inversion effects. The shaded region represents the 95% CIs around the mean inversion effect for each subject.

Our initial goal was to quantify the per-subject individual differences in holistic processing of Mooney faces. We operationalized the extent to which a subject processed Mooney faces holistically as the average magnitude of the inversion effect (average accuracy in the upright condition minus the average accuracy in the inverted condition) across all Mooney faces (Figure 3). We found that the magnitude of the inversion effect varied significantly across subjects (Figure 3B). The individual differences found here replicate prior work (Russell et al., 2009; Wang et al., 2012).

**Figure 4:**
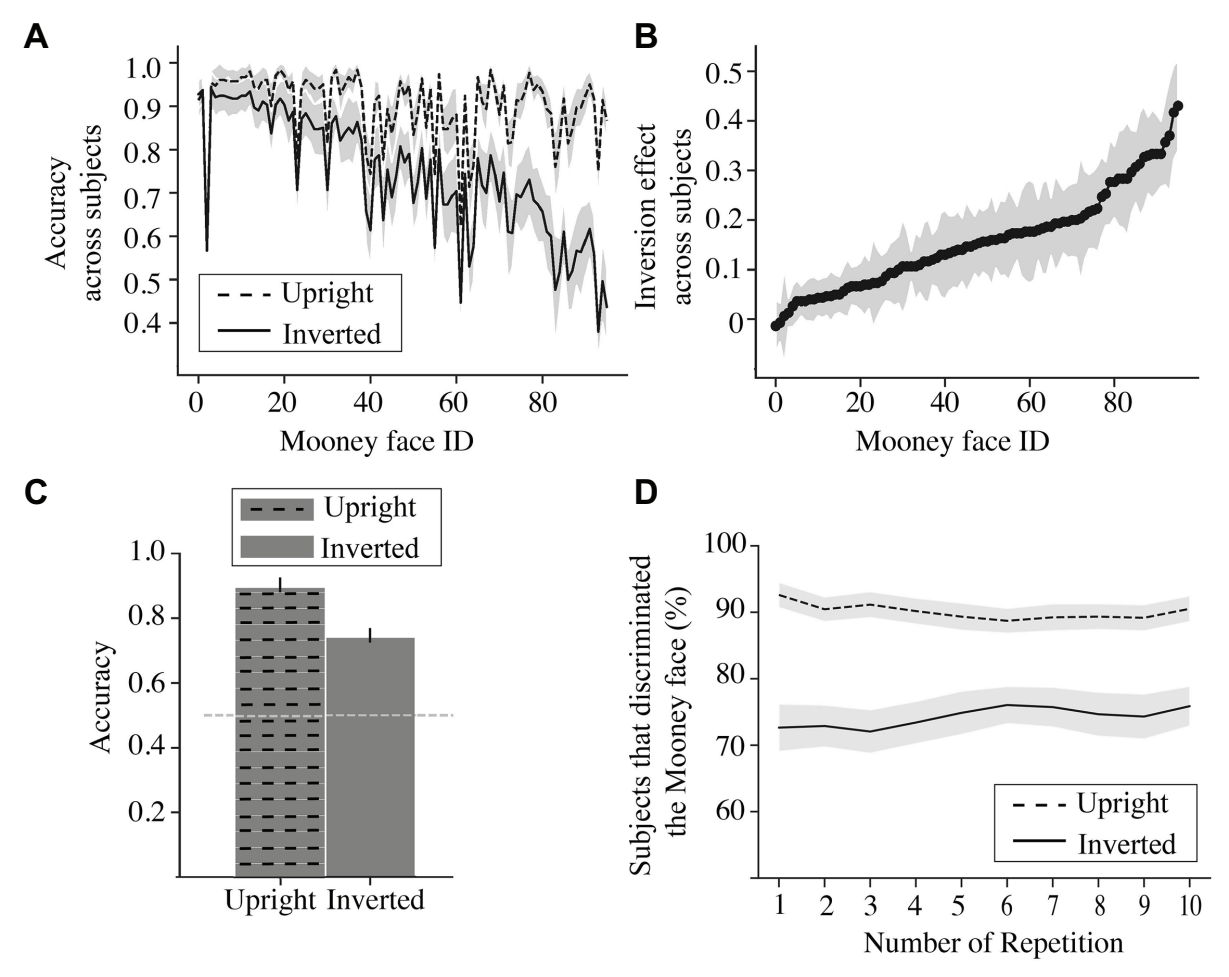
Experiment 1 results. **(A)** Upright (dashed) and inverted (solid) accuracy for each Mooney face. Results for each Mooney face (abscissa) are averaged across subjects. The shaded region represents the 95% CI. **(B)** Magnitude of the inversion effect (difference between accuracy in upright condition and accuracy in inverted condition from panel **A**) for each Mooney face (abscissa). The shaded region represents the 95% CI of the mean across subjects. **(C)** Average accuracy across subjects and across Mooney faces for upright (dashed) and inverted (solid) conditions. The error bars represent the 95% CI of the mean across subjects and Mooney faces. The gray dashed line represents the 50% at chance accuracy. **(D)** Percentage of subjects that successfully discriminated the Mooney face at each repetition of Mooney face (abscissa). Shaded area represents the 95% CIs of the mean across Mooney faces.

Our primary goal was to investigate the *per-face* differences in holistic perception for individual subjects. That is, are the individual differences stimuli specific? To address this, we calculated the magnitude of the inversion effect for each Mooney face across subjects. We found that individual Mooney faces varied significantly in the magnitude of their inversion effect (Figure 4B) and these differences reflected changes in performance in both upright and inverted conditions (Figure 4A). That is, not all Mooney faces tapped into holistic processing to the same extent.

As a simple confirmation of the previously documented inversion effect (Yin, 1969), we also calculated the average accuracy collapsed across Mooney faces and subjects for the upright and inverted conditions, separately. Replicating the extensively studied inversion effect, we found higher accuracy for upright (Mean = 0.90; *SD* = 0.016) than for inverted faces (Mean = 0.742; *SD* = 0.028) across all subjects and Mooney faces, *t*(95) = 9.618, *p* < 0.001 (Figure 4C).

In addition, we tested whether subjects were consistent in recognizing the Mooney faces across their 10 trials (Figure 4D). We found that if recognized in the first instance, the same upright Mooney face image was also recognized in subsequent presentations as well (Figure 4D, slope parameter of linear regression *b* = −0.07, *p* = 0.05). Conversely, if the Mooney face was not recognized in the first trial, it was also not recognized in subsequent trials. Subjects did get slightly better at recognizing inverted Mooney faces, but the effect was modest (Figure 4C; slope parameter of linear regression, *b* = 0.11, *p* < 0.05).

**Figure 5:**
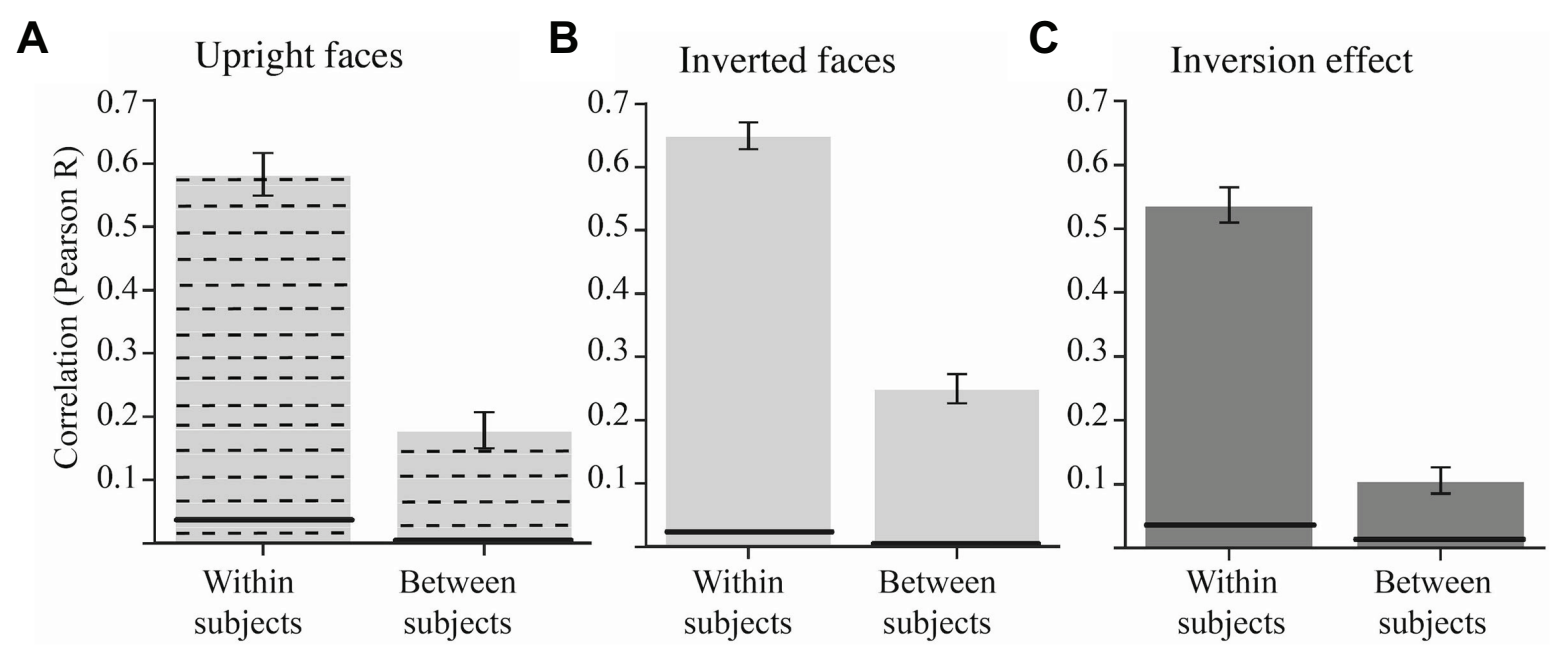
Average within‐ and between-subject agreement in the accuracy scores for the Mooney face task across subjects for the upright condition **(A)**, the inverted condition **(B)** and the inversion effect **(C)**. The within-subjects agreement is calculated using a bootstrapped split-half correlation (more details in the Results section). The error bars represent the 95% CI of the bootstrapped mean. The black line in each bar represents the upper 97.5% upper bound of the bootstrapped null distribution.

So far, we replicated the extensive literature showing that subjects differed in the extent they process faces holistically and we extended these results by showing that not all Mooney faces tap into holistic processing to the same extent. Next, we wanted to investigate whether subjects agreed on which Mooney faces are processed holistically. To that end, we investigated the between-subject agreement in the magnitude of the inversion effect for each Mooney face by calculating the mean average pairwise correlations of subjects’ per-face inversion effect. We found relatively low between-subject correlation in the inversion effect [Figure 5C; *r* = 0.106, CI = (0.085, 0.127)], although it remained significantly different from zero (*p* < 0.001). Interestingly, the low between-subject agreement reveals that subjects do not process Mooney faces holistically to the same extent (Figure 5C). In fact, we found that there was a low between-subject correlation in both upright and inverted accuracies (Figures 5A,B). This result suggests that the low between-subject correlation in the inversion effect is driven by low between subject agreement in both the upright and inverted conditions.

To quantify the consistency of these per-face individual differences in holistic processing, we also calculated the within-subject agreement for each Mooney face. The within-subject correlation was performed using a split-half procedure. That is, for each subject and each Mooney face, we separated the upright and inverted trials in two halves (bootstrap with resampling) and then also calculated each face’s inversion effect for each half separately. The within-subject agreement was the correlation between the inversion effects of these two halves. We found that there was a high within-subjects correlation in the inversion effect [Figure 5C; *r* = 0.537, CI = (0.510, 0.563), *p* < 0.001]. The high within-subjects correlation shows that subjects were consistent within themselves about the degree to which any particular face was holistically processed (Figure 5C). The same pattern of results was found for upright and inverted discrimination (Figures 5A,B). This high within-subject consistency reveals that the low between-subject correlation found in Figure 5 was not due to noise.

Lastly, the difference between the within-subjects and between-subjects correlation in the inversion effect was statistically significant (*p* < 0.001, based on bootstrap test). That is, we found consistent per-face individual differences in holistic processing of Mooney faces.

### Discussion

Taken together, our results reveal consistent per-subject individual differences in the extent of holistic processing of Mooney faces and stable per-face differences in the extent to which subjects process Mooney faces. Interestingly, we found that subjects do not agree on which Mooney faces are more holistically processed. That is, the Mooney faces that are processed more holistically are not the same for all subjects. These differences are stable within-subject, as reflected by the high within-subject correlations. These idiosyncratic per-face individual differences in holistic processing between subjects do not originate only from individual differences in perceiving the inverted faces.

## Experiment 2: an Operational Definition of Mooney Face “Holistic-Ness”

Experiment 1 revealed individual subject and per-face differences in holistic processing of Mooney faces. Although observers had stable holistic representations of the Mooney faces within themselves, subjects varied significantly in the extent to which they process Mooney faces holistically. Additionally, Mooney faces showed significantly different levels of holistic processing across subjects. These results suggest that Mooney faces are not all equally good at isolating holistic processing and that some Mooney faces are easier to recognize than others. Experiment 1 showed that there are individual differences in Mooney face perception that operate at the level of particular faces. Since Mooney faces seem to vary in their difficulty and the extent they tap into holistic processing, it is possible that the lack of between-subjects agreement is limited to harder Mooney faces that may require observer-specific high-level representations. To explore this hypothesis, in Experiment 2, we first proposed two ways to identify the harder Mooney faces that required relatively more holistic processing. One way is to use the magnitude of the inversion effect for each Mooney face, which we already calculated in Experiment 1. Another way may be to examine the contours (the outlined edges) of the Mooney faces. Typically, the outlines of Mooney faces are believed to be unrecognizable (Moore and Cavanagh, 1998), but this has not been systematically examined in the literature. Harder Mooney faces that most effectively tap into holistic processing should lack segmentable parts and their contours should therefore be harder to recognize. In contrast, faces that depend on part-based processing might have contours that are easier to recognize. In sum, Experiment 2 aimed to test the hypothesis that the per-face idiosyncratic holistic perception exists for both harder and easier Mooney faces.

### Method

#### Participants

Twenty-three participants took part in this experiment. All subjects were undergraduate students at the University of California, Berkeley and provided written consent form before participation. All experimental procedures were approved by the UC Berkeley Institutional Review Board.

#### Materials

**Figure 6:**
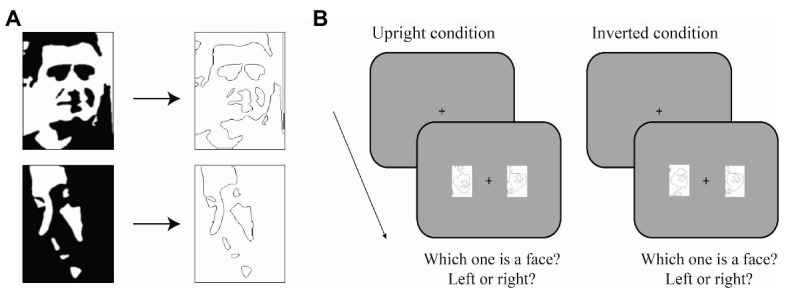
Experiment 2 example stimuli and design. **(A)** Two examples of Mooney faces and their respective outlines. **(B)** Procedure in the outline condition. The left and right panels represent the upright and inverted conditions, respectively. The full Mooney condition is not depicted. Stimuli, task, and procedure were exactly the same as in Experiment 1.

The Mooney face stimulus is the same as in Experiment 1. An edge-detection algorithm with Sobel-method was run on each of the 192 Mooney face stimuli (scrambled stimuli included). Figure 6A illustrates an example of two Mooney faces and their respective output outline after running the edge-detection algorithm on them.

#### Design

About two edge conditions (full Mooney vs. outline) × 2 Orientation conditions (upright vs. inverted) × 96 Mooney stimuli × 4 repetitions of each Mooney Face. Subjects ran a total of 1,536 trials.

#### Procedure

Task and procedure were the same as in Experiment 1, with the exception that there was an extra condition: the stimuli could be either a Mooney face and a scrambled Mooney face or an outline of a Mooney face and an outline of a scrambled Mooney face. As in Experiment 1, the Mooney face (or the outline of the Mooney face in the outline condition) could be either upright or inverted. Thus, there were four conditions (full vs. outlined Mooney face × 2 orientations). The subject’s task was always to determine which of two images was a face regardless of its orientation. The four conditions were randomly interleaved between trials and divided into 10 blocks. Figure 6B show a sample trial of the outline condition for upright and inverted faces. Trials in the full Mooney face condition were exactly the same as in Experiment 1.

### Results

Replicating the inversion effect and the results in Experiment 1, accuracies for the upright condition were overall higher than for the inverted condition in the full Mooney condition, *t*(95) = 9.54, *p* < 0.001. For both upright and inverted conditions, the accuracy was lower for the outline Mooney than the full Mooney condition, *t*(95) = −12.14, *p* < 0.001 and *t*(95) = −5.40, *p* < 0.001.

**Figure 7:**
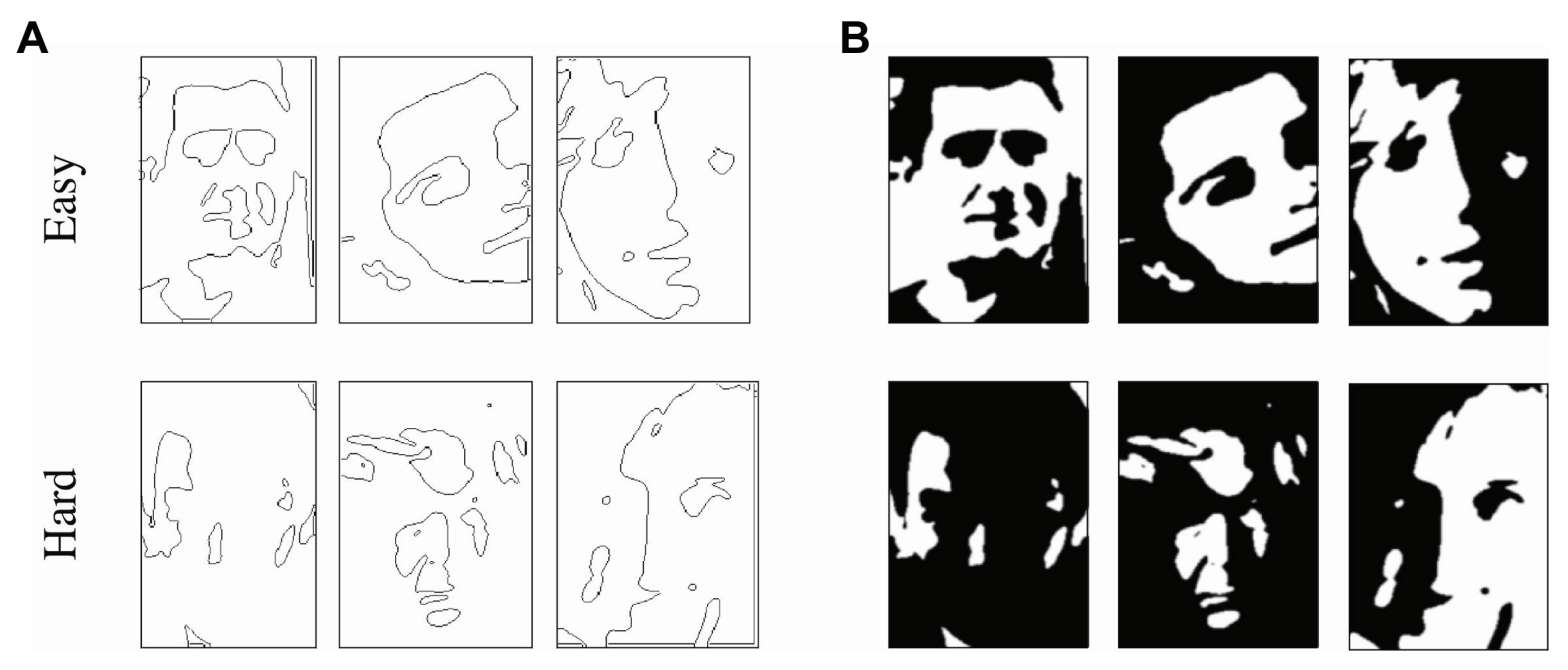
**(A)** Examples of outlines that are easy (upper row; higher accuracy in outlined Mooney condition) and hard (lower row; lower accuracy in outlined Mooney condition) and **(B)** the respective full Mooney faces.

In Experiment 2, our goal was to investigate whether the per-face individual differences in holistic processing appear in both hard and easy Mooney faces. A hard Mooney face would be one that lacks any low-level, single face features, which means that it would show a strong inversion effect and its outline should be unrecognizable. The per-face inversion effects were pulled from Experiment 1. Here, we used the accuracy in the outlined Mooney condition as a measure of how recognizable the individual features of the face are (eyes, nose, mouth, etc.). The results indicated that some Mooney outlines were easy to recognize, and some were difficult to recognize. Figure 7A shows examples of outlined Mooney faces that were particularly easy to recognize (i.e., showed high accuracy in the outlined condition) or were particularly difficult to recognize (i.e., showed low accuracy in the outlined condition), along with their respective full Mooney versions (Figure 7B). Then, we calculated the Pearson’s correlation between the difficulty of each outlined Mooney face with the magnitude of the inversion effect for each respective face, taken from the data in Experiment 1 (that is, the two sets of data come from two separate group of subjects).

**Figure 8:**
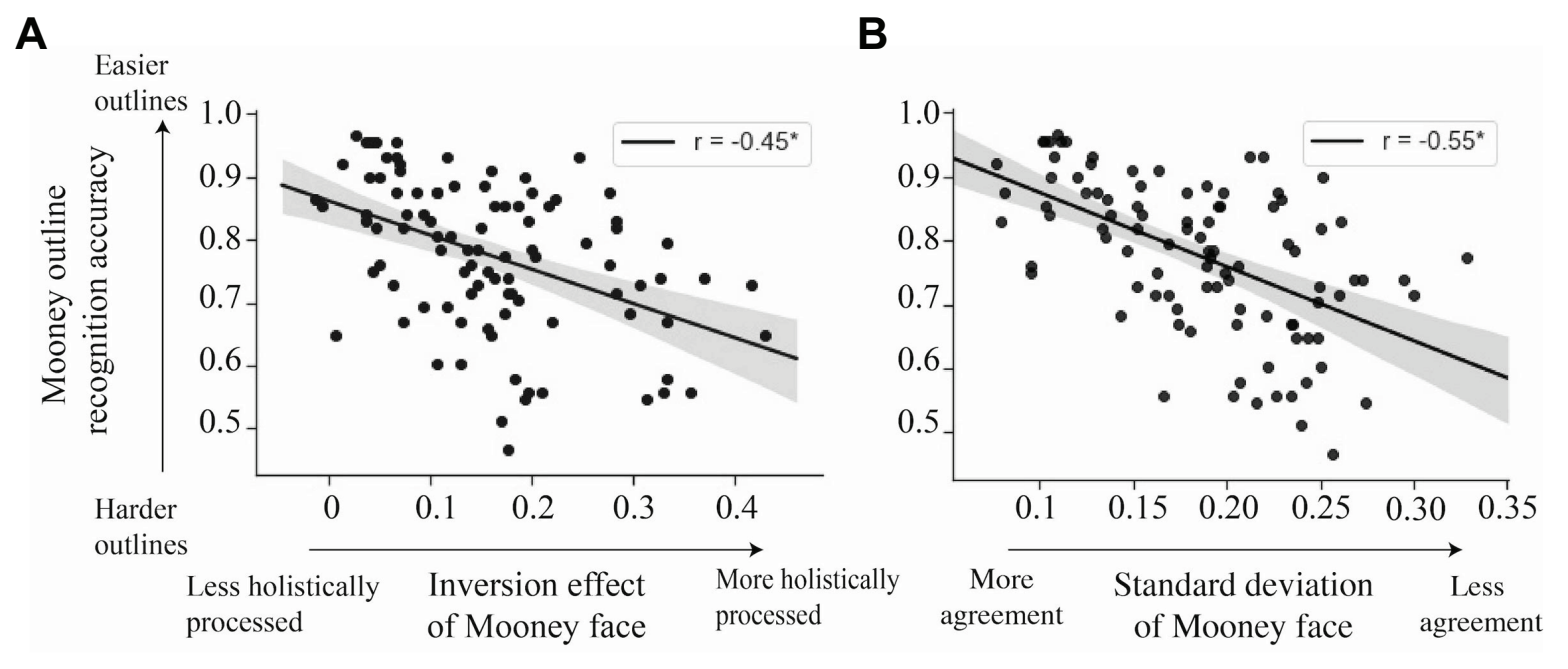
**(A)** Correlation between the magnitude of the inversion effect for each Mooney face from Experiment 1 (abscissa) and the discrimination of the corresponding upright outlined Mooney from Experiment 2. The two axes represent independently collected sets of data from different groups of observers. Each dot represents one Mooney face. The shaded region represents the 95% CIs of a linear regression. There is a correlation between the recognizability of outlines and the holistic-ness of the full Mooney faces. **(B)** Correlation between the between-subjects agreement in the inversion effect of each Mooney face from Experiment 1 (measured by SD of each Mooney face inversion effect from Experiment 1 – abscissa) and the discrimination of each upright outlined Mooney face from Experiment 2 (ordinate). There is a correlation between the recognizability of outlines and the extent to which subjects agree on the inversion effect of the full Mooney faces. Each dot represents a Mooney face. The shaded region represents the 95% CIs of a linear regression.

To test the association between the two measures – the magnitude of the inversion effect and the difficulty of detecting a face in its outline – we pulled the inversion effect values of each Mooney face from Experiment 1 and correlated them with their accuracy in the outlined condition in Experiment 2. That way, each variable came from independent subjects. We found that the variability in recognizability of the outlined Mooney faces was correlated with the degree to which the full Mooney faces required holistic processing. That is, the easier to recognize an outline was (measured by the accuracy of the outline Mooney in upright condition), the weaker the inversion effect for that Mooney face was, Pearson’s *r* = −0.45, *p* < 0.001, CI = (−0.60, −0.28; Figure 8A). These results suggest that there may be a continuum of how hard a Mooney face is to recognize and how much a Mooney face taps into holistic processing.

**Figure 9:**
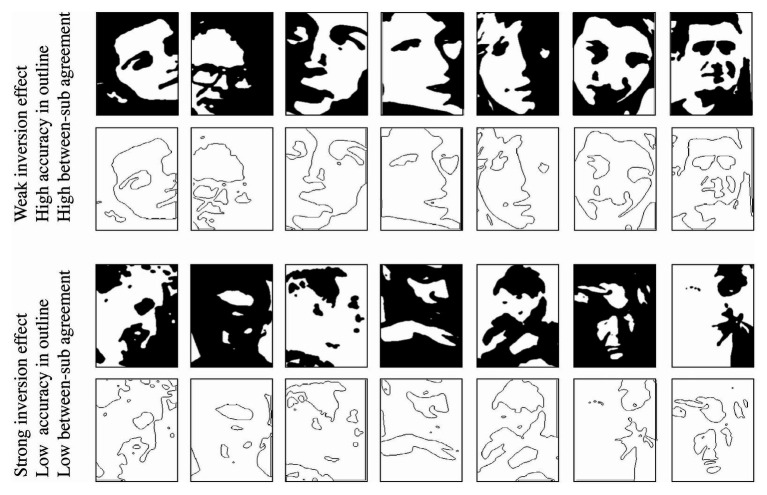
Examples of Mooney faces and outlines that differed in inversion effect, outline recognizability, and between-subject agreement. Top row: example Mooney faces with weaker inversion effect, easily recognized outlines, and high between-subject agreement. Bottom row: Mooney faces with stronger inversion effect, difficult-to-recognize outlines, and low between-subject agreement. For both rows, magnitude of the inversion effect and between-subject agreement of Mooney faces are pulled from Experiment 1, while difficulty of discrimination of outlined Mooney faces is taken from Experiment 2.

Could the per-face individual differences in holistic processing be limited only to hard Mooney faces? In Experiment 1, we found that observers did not agree on the magnitude of the inversion effect for each Mooney face. Here, we wanted to test whether the per-face, per-subject differences found in Experiment 1 are limited to those hard faces that cannot be processed by parts and require holistic processing (i.e., that have a strong inversion effect). Experiment 2 revealed that hard Mooney faces, which tap into holistic processing, can be independently identified by the recognizability of their outlines. Following on this, we correlated the outline recognizability with the level of between subject agreements in the magnitude of the inversion effect of each face. The recognizability of each outlined face was measured by the accuracy in the upright outline condition in Experiment 2. To quantify the between-subjects agreement for each Mooney face, we used the between-subject SD of the inversion effect for each Mooney face in Experiment 1. A SD close to zero represents nearly perfect agreement and the higher the SD gets, the lower the between-subject agreement. Importantly, the two sets of data calculated here – the per-Mooney-face between-subject agreement and the difficulty of each outline face – come from different subjects (from Experiments 1 and 2, respectively). Our results show a negative correlation between the accuracy in recognizing the upright outlined Mooney face and the agreement between subjects, Pearson’s *r* = −0.55, *p* < 0.01, CI = (−0.67, −0.39; Figure 8B). Faces with higher agreement have higher upright accuracy in the outline condition. Faces with less between subject agreements have outlines that are less recognizable. That is, subjects agree more on the easy Mooney faces that had more cartoon-like outlines and weaker inversion effects (Figure 9, top row). In contrast, the individual differences are more apparent in the hard faces that have stronger inversion effects and whose outlines are unrecognizable (Figure 9, bottom row).

### Discussion

Our results show that the recognizability of a Mooney face’s outline predicts the extent to which that Mooney face is processed holistically. These results confirm and extend the results, we found in Experiment 1 where each Mooney face varied in the extent to which it was holistically processed. Those Mooney outlines that are recognizable (e.g., top row, Figure 9) and are essentially seen as cartoon-like. Hard, holistic Mooney faces in the outline condition are literally outlines of blobs. Their full counterpart lacked any single feature information, so their outlines did too. Turning the hard Mooney face into an outline removed the possibility of processing them holistically, which prevents their discrimination. Contrary, outlines of easy, part-based faces maintained the single face feature information, which allowed them to be processed in a part-based manner, and possibly holistically too. Thus, the categorization of how cartoon-like the Mooney face is reveals which faces tap into holistic processing.

Importantly, the results in Experiment 2 suggest that the lack of between-subject agreement in each face’s inversion effect found in Experiment 1 is mainly driven by the faces that tap into holistic processing (i.e., have stronger inversion effect) and whose outlines are hard to recognize. In other words, subjects show a higher level of agreement only for those cartoon-like faces that have clearly segmentable features and weaker inversion effects. This echo the results of the first experiment: holistic processing seems to be very subject-specific. Studies aiming to investigate holistic processing using hard Mooney faces that cannot be processed part-by-part should take into account these individual differences.

## General Discussion

Mooney faces are frequently used to study holistic processing and its role in face recognition (Latinus and Taylor, 2005, 2006; Otsuka et al., 2012; Verhallen et al., 2014; McCaffery et al., 2018). The individual differences approach in holistic processing can help elucidate what makes our ability to process faces unique (Yovel et al., 2014). By examining each observer’s idiosyncratic way of processing faces, we can isolate the face processing mechanisms that are universally shared across humans and those that are idiosyncratic to each observer’s experience. Because Mooney face perception is subject to individual differences (Verhallen et al., 2014), and because these individual differences in holistic processing likely depend on subject-specific top-down knowledge and prior experience (Michel et al., 2006; Zhou et al., 2012), one might expect that there should be both individual *subject*-specific differences as well as individual *stimulus*-specific differences in holistic processing. Consequently, some particular Mooney faces may be easier to recognize for some particular subjects. To address this, we tested whether there are individual differences in the extent to which humans process Mooney faces holistically and whether Mooney faces vary in the extent to which they tap into holistic processing by calculating the magnitude of the inversion effect of each individual Mooney face.

Our results showed that there are individual differences in the extent to which subjects process Mooney faces holistically. This finding replicates previous studies that include not only Mooney faces as a measure of holistic processing, but also use the inversion effect in gray-scale faces (Verhallen et al., 2014; McCaffery et al., 2018), the composite-face effect (Richler et al., 2011) and the whole-part effect (Wang et al., 2012). Importantly, observers did not agree on which faces had a weak or strong inversion effect; that is, which faces were holistically processed. Yet, observers did show within-subject consistency in their holistic perception of the Mooney faces. These stable per-face idiosyncratic differences indicate that the holistic representations of Mooney faces are not universal or shared across subjects.

Furthermore, we found that upright Mooney faces were either recognized in the first repetition or not recognized at all, and once an observer recognized a Mooney face they could not “unsee” it (Brodski et al., 2015), much like Dallenbach’s cow (Dallenbach, 1951) and Gregory’s Dalmatian dog (Gregory, 1970). Note that inverted faces did show an improvement with repeated face exposures. Here, we extend these results to Mooney faces and show that the high within-observer consistency occurs for specific faces. Mooney faces, unlike other kind of faces, may require high-level representations that are observer-specific. If an observer has a template that facilitates the discrimination of a particular Mooney face, then the observer will recognize it in the first trial; otherwise, the face will not be recognized regardless of the number of repetitions. These face templates may be shaped by individual experience, which would make them unique to each observer.

Previous studies suggest that faces and objects lie on a continuum, such that faces are processed as a whole and objects are processed by their parts (Farah, 1991). The results from Experiment 1 showed that Mooney faces vary significantly in how much they tap into holistic processing. That is, not all Mooney faces are processed holistically to the same extent. Our results suggest that given a Mooney face stimulus set, one can filter out which faces can be recognized using feature-based processing by independently measuring the recognizability of the outlines. Several research groups have created Mooney faced datasets by either directly drawing them (Mooney, 1957), thresholding grayscale faces (Verhallen and Mollon, 2016; Schwiedrzik et al., 2018) or generating them through machine learning algorithms (Ke et al., 2017). However, none of them include an evaluation of the “holistic-ness” of the Mooney faces.

The approach developed here shows that one can use the contour of the face to predict how holistic it is on a group or even individual subject basis. Although Mooney contours are often considered *ambiguous* (Moore and Cavanagh, 1998), our results show that some Mooney faces do have recognizable contours. Many Mooney outlines are completely unrecognizable, but some appear cartoon-like. The cartoon-like Mooney outlines were associated with easily recognized Mooney images. Conversely, the outlines that were unrecognizable were derived from Mooney faces that required more holistic processing. Using the technique here and measuring outline recognizability can help future studies isolate holistic processing by selecting the most appropriate Mooney faces.

Importantly, quantifying the “holistic-ness” of the Mooney faces using this approach revealed that per-face, per-observer differences in holistic processing only occurred for hard Mooney faces that required holistic processing. Conversely, discrimination of easily segmentable Mooney faces was shared across subjects and seems to enjoy more universal agreement. These findings suggest that holistic processing of hard faces depends on a particular observer’s experience or idiosyncratic templates, whereas processing of easy faces proceeds similarly across observers. Top-down guidance, upon which holistic face perception of hard faces may rely, depends on knowledge and prior experience. Since knowledge and prior experience is unique to the observer, the individual differences are more apparent in those faces that are harder to recognize and require more holistic processing. Altogether, these results support the conclusion that holistic and part-based processing are distinct mechanisms of face recognition (Moscovitch et al., 1997; McKone, 2004). Here, we provide further evidence for a dissociation between these two mechanisms: part-based processing is universal, while holistic perception is individual-specific and depends on experience.

A large number of behavioral and neuroimaging studies have aimed to understand holistic perception and its role in face recognition. Many of these studies use Mooney faces and presume that holistic processing is involved (Kanwisher et al., 1998; Andrews and Schluppeck, 2004; George et al., 2005; Verhallen et al., 2014; Verhallen and Mollon, 2016; McCaffery et al., 2018). Some of this previous work has taken into consideration the idea of individual differences in the extent to which Mooney faces are recognized (Andrews and Schluppeck, 2004; George et al., 2005), in agreement with our results. However, Mooney face awareness was collapsed across observers in these studies, which did not allow for a further analysis of the stability of these differences or the possible stimulus-specific individual differences. Our results show that the degree to which a Mooney face is processed holistically is idiosyncratic: it depends on the particular face and the particular observer. Therefore, to really understand the nature of holistic representations, future research should take this idiosyncrasy into account.

## Data Availability Statement

The raw data supporting the conclusions of this article will be made available by the authors upon request, without undue reservation.

## Ethics Statement

The studies involving human participants were reviewed and approved by UC Berkeley Institutional Review Board. The patients/participants provided their written informed consent to participate in this study. Written informed consent was obtained from the individual(s) for the publication of any potentially identifiable images or data included in this article.

## Author Contributions

Both the authors contributed to the study concept and the study design. TCB programmed the software and performed data collection and data analysis. The manuscript was drafted by TCB and was reviewed and edited by DW. The figures were made by TCB. Both the authors contributed to the article and approved the submitted version.

### Conflict of Interest

The authors declare that the research was conducted in the absence of any commercial or financial relationships that could be construed as a potential conflict of interest.
